# Lateral Prefrontal Cortex Mediates the Cognitive Modification of Attentional Bias

**DOI:** 10.1016/j.biopsych.2009.10.031

**Published:** 2010-05-15

**Authors:** Michael Browning, Emily A. Holmes, Susannah E. Murphy, Guy M. Goodwin, Catherine J. Harmer

**Affiliations:** Department of Psychiatry, University of Oxford, Warneford Hospital, Oxford, United Kingdom

**Keywords:** Anxiety, attention, cognitive bias, cognitive training, emotion, fMRI

## Abstract

**Background:**

A tendency to orient attention toward threatening stimuli may be involved in the etiology of anxiety disorders. In keeping with this, both psychological and pharmacological treatments of anxiety reduce this negative attentional bias. It has been hypothesized, but not proved, that psychological interventions may alter the function of prefrontal regions supervising the allocation of attentional resources.

**Methods:**

The current study examined the effects of a cognitive training regime on attention. Participants were randomly assigned to one of two training conditions: “attend-threat” training, which increases negative attentional bias, or “avoid-threat” training, which reduces it. The behavioral effects of training were assessed using a sample of 24 healthy participants. Functional magnetic resonance imaging data were collected in a further 29 healthy volunteers using a protocol that allowed the influence of both stimuli valence and attention to be discriminated.

**Results:**

Cognitive training induced the expected attentional biases in healthy volunteers. Further, the training altered lateral frontal activation to emotional stimuli, with these areas responding specifically to violations of the behavioral rules learned during training. Connectivity analysis confirmed that the identified lateral frontal regions were influencing attention as indexed by activity in visual association cortex.

**Conclusions:**

Our results indicate that frontal control over the processing of emotional stimuli may be tuned by psychological interventions in a manner predicted to regulate levels of anxiety. This directly supports the proposal that psychological interventions may influence attention via an effect on the prefrontal cortex.

Anxious individuals are exquisitely sensitive to distraction by mildly threatening stimuli (1–3). A range of evidence suggests that this negative attentional bias may be a causal factor in generating and maintaining anxiety rather than simply being an epiphenomenon of the anxious state. Most convincingly, a number of recent studies have used a cognitive training paradigm to alter attention to emotional stimuli and have been able to demonstrate experimentally that inducing a negative attentional bias in healthy participants increases anxiety (4), while reducing negative attentional biases in clinically anxious populations improves anxiety (5,6). Similarly, administration of selective serotonin reuptake inhibitors, which are effective in the treatment of a range of anxiety disorders (7), has been found to reduce negative and increase positive attentional bias in nonclinical groups (8,9). There is thus evidence that negative attentional bias is causally linked to the symptoms of anxiety and that these biases can be altered using either pharmacological or psychological strategies.

Neural models (10–12) of attentional control suggest that two biasing signals influence the deployment of attention to emotional stimuli. An amygdala based system produces a signal that automatically promotes the deployment of attention toward salient stimuli. A more flexible response is associated with a second signal, originating in areas of the prefrontal cortex (PFC) (including the rostral anterior cingulate cortex [rACC] and the lateral prefrontal cortex [lPFC]), and is evoked when conflicting demands are made on attention (13,14). Both kinds of biasing signal are thought to harness processing resources in the sensory and association cortices in favor of their preferred, and at the expense of their less preferred, stimuli. In neural terms, increased attention to a stimulus, generated by either the amygdala or prefrontal cortical system, is associated with increased activation of the relevant sensory and association cortices in response to that stimulus (15). Interventions that modify emotional attention may thus plausibly be mediated by alteration of the function of either the amygdala or the prefrontal biasing signals; the effects of the interventions on attention would be predicted to be reflected in altered sensory and association cortex activation to emotional stimuli.

Direct experimental evidence indicates that antidepressant medications reduce amygdala activation to threatening stimuli and increase visual association cortex response to positive stimuli (16–21), suggesting that these drugs may alter attentional habit via an effect on early stimulus appraisal rather than on higher order control processes. It has been suggested that psychological treatments, in contrast, are likely to work through changes in the frontal control systems (22,23). While this seems plausible, the complexity and variability of formal psychotherapies such as cognitive-behavioral therapy (CBT) complicate the interpretation of their effects in controlled experimental trials. It appears more logical to study experimentally the mechanisms of their component procedures. Using this approach, explicit methods of emotional reappraisal have been demonstrated to be associated with alteration in prefrontal function (24). However, there is little evidence regarding the mechanisms by which habitual attentional bias may be influenced. Accordingly, we have investigated the mechanisms by which a computerized cognitive training task (25) alters attentional bias using both behavioral measures and blood oxygenation level-dependent (BOLD) functional magnetic resonance imaging (fMRI) signal. Consistent with our previous work, which examines the mechanisms of pharmacological interventions (8,17,20,26,27), a nonclinical sample was used in the current study. This strategy allows us to investigate the direct effects of the cognitive intervention on neural processing and behavior, unconfounded by the mood changes that can accompany such interventions in clinical populations. We hypothesized that attentional training would induce a bias in attention that we could measure behaviorally and that this would primarily be mediated by alteration of rACC and lPFC functions. We predicted that these changes in frontal function would be associated with secondary changes in visual sensory association cortex (22,23). We assayed the changes in the frontal control regions that are produced by attentional training by placing subsequent conflicting demands on attention (13,14); we specifically predicted that response in these areas would be greatest during trials in which the direction of participants' attention conflicted with their training and least when it conformed with it. Our findings support the hypothesis that the frontal cortex mediates the attentional effects produced by psychological treatment.

## Methods and Materials

### Participants

A total of 53 native English-speaking, healthy participants were randomly assigned to either “attend-threat” or “avoid-threat” training conditions (see Attentional Training Task below). All participants provided written informed consent to the study, which had been approved by an Oxfordshire Research Ethics Committee. Immediately following the training task, 24 participants (12 in each group) completed a behavioral assessment of the training procedure. In the remaining 29 participants (attend-threat = 14, avoid-threat = 15), the effects of training were assessed using an fMRI paradigm. Independent samples were used to assess the different outcome modalities (behavior and BOLD response), as completion of either assessment task would be predicted to reduce the strength of the attentional training effect; the current design was therefore intended to be maximally sensitive by allowing both behavior and BOLD response to be assessed immediately following training. Participants were screened to exclude current or previous Axis I psychiatric disorders or alcohol/substance misuse using the Structured Clinical Interview for DSM-IV (28). Participants were also excluded if they were taking any psychoactive medication, had any significant neurological condition, or were familiar with any of the tasks or stimuli used in the study. All participants who completed fMRI scanning were right-handed.

### Questionnaire Measures

Participants completed questionnaire assessments of depressive (Beck Depression Inventory) (29) and anxious symptomatology (trait subscale of the State-Trait Anxiety Inventory) (30). State anxiety and mood were also assessed before and after completion of the training task (using both the state subscale of the State-Trait Anxiety Inventory and visual analogue scales measuring happy, sad, anxious, and relaxed) to monitor whether the training task induced any changes in mood.

### Attentional Training Task

**Figure 1:**
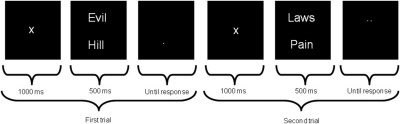
Example trials from the attentional bias training task. Two words were presented, one above the other, on a computer screen. After 500 msec, the words were replaced by a probe (a single dot or two dots) in the location of one of the words. The participants were instructed to respond by button press to indicate whether the probe consisted of one dot or two. The word pairs used were taken from the study by MacLeod *et al.* (4) and consisted of a negative word (e.g., pain) and a neutral word (e.g., laws). Attentional training was achieved by controlling the position of the probes such that in the avoid-threat group the probes were always in the position of the neutral word, whereas in the attend-threat group, the probes were always in the location of the negative word. The training task consisted of a total of 576 trials in pseudorandom order, as well as three rest sessions. The figure illustrates two trials from the avoid-threat training condition in which the probes always replaced the neutral word. The attend-threat training condition was identical in every respect other than that the probes replaced the threatening words.

The attentional bias training procedure (Figure 1) replicated the method described by MacLeod *et al.* (4). Over the course of training, participants learn to attend to the valence of stimuli that predict the location of the probe to which they have to respond; therefore, the attend-threat training encourages a negative attentional bias, whereas the avoid-threat training encourages a tendency to avert attention from negative stimuli.

### Behavioral Assessment

The effects of the training task on a behavioral measure of attentional bias were assessed using a version of the dot-probe task (31). The pertinent differences between this task and the training task were that pictures of faces displaying fearful or neutral expressions were used in the place of word stimuli (32,33) and the probe had an equal probability of replacing the fearful or neutral face. Because the emotional intensity of facial stimuli has been shown to influence measures of attentional bias (34), morphing software was used to combine the fearful with neutral expression to create a range of fearful intensities (100%, 75%, 50%, 25%, 0% fearful expression). Each intensity was presented on 20 occasions giving a total of 100 trials.

### Imaging Task

**Figure 2:**
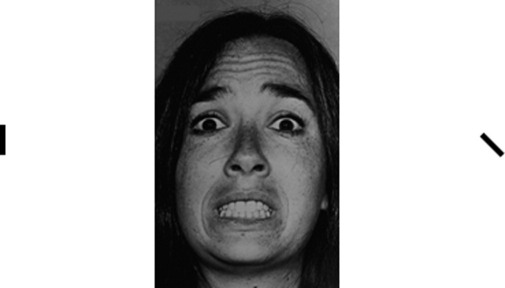
Behavioral task completed during the scan, example trial (35). Following a centrally presented fixation cross, a picture of a face (reproduced with permission from [47]) flanked by two bars was presented for 200 msec. Manipulation of the affective quality of the stimuli was achieved by presenting either fearful (shown) or neutral faces (only 100% fearful faces were used in scanning, as it was at this intensity that the maximal behavioral effect was found). The direction of attention of participants was manipulated using sequential blocks of 20 trials during which participants were instructed to respond by button press to either the gender of the face (i.e., requiring that attention is focused on the face) or to whether the flanking bars were aligned (i.e., requiring that attention is directed away from the face). The overall structure of the task was thus factorial with two levels of emotion (fear and neutral) and two levels of attention (toward and away from the faces). Participants had a maximum of 4 sec to make a response, after which there was a jittered intertrial interval (jitter was created using an exponential function resulting in an ISI ranging from a minimum of 6 sec to a maximum of 12 sec). In total, eight blocks were completed per subject, leading to 160 trials. The task took approximately 20 min to complete. ISI, intertrial interval.

The effects of training on neural activity were assessed with a task (Figure 2) that was adapted from Pessoa *et al.* (35). Importantly, this task is behaviorally insensitive, allowing interpretation of the neural findings unconfounded by differences in behavior between groups.

### Image Acquisition

A BOLD contrast signal was acquired using echo planar imaging on a 3T Siemens TIM Trio System (Siemens, Erlangen, Germany). A total of 45 slices were acquired using a voxel resolution of 3 × 3 × 3 mm^3^, repetition time = 3 sec, echo time = 30 msec, flip angle = 87°. The slice angle was set to 30°. The T1-weighted structural images were acquired for subject alignment using a magnetization prepared rapid acquisition gradient echo sequence with the following parameters: voxel resolution 1 × 1 × 1 mm^3^, echo time = 4.7 msec, repetition time = 2040 msec.

### Data Analysis

#### Questionnaire Data

Baseline measures were compared between groups for each part of the study using independent *t* tests for continuous data and chi-square tests for categorical data. Change in anxiety over time was assessed using a (2 × 2) split-plot analysis of variance (ANOVA) with the between-subject factor of training group and the within-subject factor of time of assessment (i.e., before or after training).

#### Behavioral Data

Median reaction time data from accurate trials on the dot-probe task were used to calculate a vigilance score by subtracting the reaction time when the probe replaced the fearful face from the reaction time when the probe replaced the neutral face (25). This produces an estimate of the attentional bias: a more positive number indicates a greater tendency to direct attention toward the fearful face (a greater negative attentional bias). Vigilance scores for each intensity of fearful face (100%, 75%, 50%, 25%) were entered into a (2 × 4) split-plot ANOVA with training group as the between-subject factor and emotional intensity of the fearful face as the within-subject factor.

#### Image Analysis

Functional magnetic resonance imaging analysis was carried out using the default options (Methods and Materials in Supplement 1) of FMRI Expert Analysis Tool Version 5.91 (part of the Functional Magnetic Resonance Imaging of the Brain Software Library, Oxford Centre for Functional Magnetic Resonance Imaging of the Brain, Oxford University, Oxford, United Kingdom, http://www.fmrib.ox.ac.uk/fsl). As we had hypothesized that the training effect would be mediated by alteration of frontal function, we were interested in identifying regions in which activity was greatest when the task conflicted with the training participants had received. Activity in these regions should be highest in the avoid-threat group when they were attending toward the fearful or away from the neutral face (as their training had encouraged the opposite tendency). Activity in the attend-threat group should mirror this, as exactly the opposite trials would conflict with their training. This pattern of activity is captured by an interaction contrast (emotion × attention) that was constructed at the individual level from the four basic trial types of the behavioral task (Figure 2) and was registered to the Montreal Neurological Institute 152 template using affine transformation. The individual contrast images were combined at the group level in a random effects analysis allowing comparison between groups. Results from this analysis were corrected for multiple comparisons across the whole brain, again using the FMRI Expert Analysis Tool Version default options. Specifically, a cluster-based correction (36) with an initial threshold of *Z* = 2.3 followed by correction over the whole brain using a significance level of *p* < .05 was used.

#### Connectivity Analysis

Having identified potential control regions in the main analysis, we went on to test whether these regions did indeed influence a neural measure of attention: activity in face selective visual sensory association cortex (the fusiform face area [37]). This was achieved using a targeted psychophysiological interaction (PPI) analysis (38) to assess the connectivity between control and sensory regions. Briefly, as the attend-threat training increases negative attentional bias, control regions should act to increase the sensory response to fearful faces, whereas following the avoid-threat training, the control regions should favor neutral faces. We therefore assessed whether the observed connectivity between control and sensory regions would produce this effect (Methods and Materials in Supplement 1). Our analysis resulted in four estimates of connectivity per participant: one each for the links between both left- and right-sided attentional control regions with left- and right-sided sensory target regions. These data were entered into a (2 × 2 × 2) split-plot ANOVA with training group as a between-subject factor and control region (left vs. right) and target region (left vs. right) as within-subject factors.

## Results

### Baseline Characteristics

There were no significant differences between groups on any of the baseline measures, indicating that randomization had been successful (Table 1). Further, there were no between-group differences on measures of anxiety or mood across training, indicating that the effects of the training cannot be attributed to a mood induction effect (all *p* > .13).

### Behavioral Data

**Figure 3:**
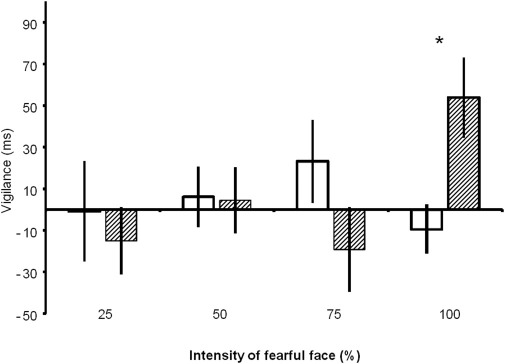
Effects of attentional training on the faces dot-probe task, a behavioral measure of attention. White = avoid-threat group, gray = attend-threat group. Intensities of fearful face (25%, 50%, 75%, and 100%) are arranged on the x axis (Error bars = SEM, *p < .05). The y axis reports the vigilance score, which is calculated by subtracting the median reaction time when the probe replaces the fearful face from the reaction time when the probe replaces the neutral face. A larger, positive vigilance score indicates a greater attentional bias toward fearful faces.

A significant group × intensity interaction [*F*(3,66) = 3.18, *p* = .03] indicated that attention training using word stimuli induced an attentional bias when assessed using faces and that this effect depended on the intensity of the facial expression. As can be seen from Figure 3, this was the result of a significant effect of training when assessed using prototypical (100%) pictures of fear [*t*(22) = 2.93, *p* = .032 (corrected for multiple comparisons)]. No significant effects of training were evident at the lower intensities of facial expression [*t*(22) < 1.5, *p* > .5 (corrected)]. Importantly, the training effect was in the expected direction, with the attend-threat group showing a greater negative bias than the avoid-threat group.

### Imaging Data

**Figure 4:**
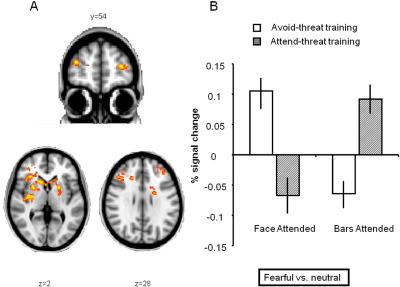
Effect of attentional training on BOLD signal. **(A)** Whole brain, cluster corrected (*Z*-threshold = 2.3, *p* < .05) analysis demonstrating bilateral frontal and striatal regions in which activity corresponded to the effects of attentional training on the emotion × attention interaction. The activation map has been rendered onto the standard MNI brain. **(B)** The mean (SEM) percent signal change associated with the fear versus neutral face contrast extracted from the right lateral PFC cluster (other clusters show an identical pattern). Estimates for the fearful face-neutral face contrast are displayed separately for trials in which participants had to attend to the location of the face (face attended) or to the location of the bars (bars attended). In all clusters, activation is greatest when participants direct their attention contrary to their training; thus, the avoid-threat training group (white bars), who have been trained to look away from threatening and toward neutral stimuli, show increased activation when looking toward threatening and away from neutral stimuli. The attend-threat training group (gray bars) show the opposite pattern of activation. BOLD, blood oxygenation level-dependent; MNI, Montreal Neurological Institute; PFC, prefrontal cortex.

Consistent with the proposal that the attentional effects of training are mediated by alteration of frontal function, whole-brain analysis comparing the emotion × attention contrast between groups revealed bilateral lPFC clusters, including dorsolateral (x y z = 36 54 16, *Z*-max = 3.22, *p*-corrected = .049) and ventrolateral PFC (x y z = 30 24 –2, *Z*-max = 3.4, *p*-corrected = < .0001) on the right and dorsolateral PFC (x y z = −30 54 10, *Z*-max = 3.27, *p*-corrected = .03) on the left (Figure 4A). Importantly, these clusters include voxels that lie within the regions of interest identified in previous studies of attentional control (13). Additionally, clusters were found bilaterally in the striatum (left: x y z = −20 6 0, *Z*-max = 3.55, *p*-corrected = .0002; right: x y z = 28 8 4, *Z*-max = 3.85, *p*-corrected = < .0001).

As these clusters had been identified using an interaction contrast, we next characterized the nature of the interaction by extracting individual estimates of the average signal change associated with fearful versus neutral stimuli separately for trials in which attention was directed toward or away from the face. All clusters revealed an identical pattern of activation (Figure 4B); we report results from the extensive right lPFC cluster, which spanned both dorsolateral and ventrolateral PFC, to illustrate this pattern. As predicted, across both training groups and all experimental trials, activity in these control regions is greatest when the direction of participants' attention conflicts with their training. Considering first the trials in which participants' attention is directed toward the faces (away from the bars), the attend-threat group has been trained to look toward negative stimuli and lPFC activation increases when they do the opposite, that is, look toward the neutral faces [compared with fearful; *t*(13) = 2.34, *p* = .036). In contrast, the avoid-threat group, whose training induced the opposite tendency, show greater activation to the fearful faces [*t*(14) = 5.25, *p* < .001]. During the trials in which participants look away from the faces (toward the bars), the attend-threat group, who have been trained to look away from neutral stimuli, show greater activity when the face is fearful [compared with neutral; *t*(13) = 4.04, *p* = .001]. Again, the avoid-threat group displays the opposite pattern of response with greater activation when neutral faces are to be avoided [*t*(14) = 3.32, *p* = .005]. Thus, lateral PFC activity is determined by two factors: the behavior of participants (as reflected in the type of information they are attending to) and the training undertaken. Across all trials and both training groups, lateral PFC activity is greatest when the participants behave contrary to their training.

Although we had predicted that the rACC would also be involved in mediating the effects of training, no activation was apparent on whole-brain analysis. However, a small cluster with a similar pattern of activation was found in the rACC when a region of interest (ROI) approach was used. Consistent with our prediction that attentional training would primarily be driven by alteration of frontal function, no such effect was apparent in the amygdala, even when using an ROI analysis (Supplement 1). As intended, the groups did not differ on performance of the task in the scanner, with equivalent reaction times and error rates (all *p* > .1).

### Connectivity Analysis

If, as predicted, the lPFC is mediating the attentional effects of training, then activity in the identified frontal regions should influence activation of the face selective visual sensory cortex (11). Specifically, in the avoid-threat group, lPFC activity should favor the sensory representation of the neutral faces, whereas in the attend-threat group, the fearful faces should be favored. Our PPI analysis tested whether the observed pattern of connectivity between the lateral frontal clusters and face selective visual sensory cortex would result in this effect. The expected pattern of connectivity was seen across both groups of participants [*F*(1,27) = 2.45, *p* = .045]. This was not modified by group, control region (left or right lPFC), target region (left or right sensory cortex), or any interaction of these factors (all *p* > .12; Methods and Materials in Supplement 1). These results are therefore consistent with our hypothesis that the information coded in lPFC activity is used in the control of attention to the facial stimuli in that the observed pattern of connectivity is consistent with that predicted by the behavioral effects of attentional training. No further clusters of activation were identified in analyses of the PPI regressors across the whole brain, and there were no significant interactions between lPFC and the amygdala when using a ROI approach.

## Discussion

The current study provides the first experimental evidence that attentional bias training can modify neural systems known to be involved in the control of attention to emotional stimuli (13,39). Specifically, lateral PFC activity depended on the type of attentional training undertaken (attend-threat or avoid-threat) and, across all participants, was greatest when the direction of participants' attention was contrary to their training. Connectivity between the identified lateral PFC clusters and face selective sensory cortex was consistent with that predicted by the behavioral effects of training and current models of selective attention (11). These results are in line with the prediction (22,23) that pharmacological (16–18,20,21) and psychological interventions that alter attentional function are mechanistically distinct.

While our main analysis showed that attentional training modulated activity in the lateral prefrontal cortex in an attentional task, it could not directly test whether these regions were actually involved in attentional control. It is conceivable, for example, that the training effect is encoded elsewhere in the brain and that the increased lPFC activity observed when the training rules were violated arise because behaving contrary to training is less practiced and thus more effortful, in essence, a form of task switching effect (40). By this interpretation, altered lPFC activity results as a consequence of training rather than mediating its effect. We therefore sought to test our interpretation of the results by examining the pattern of connectivity between the identified lateral PFC regions and face selective visual sensory association cortex. In this analysis, we reasoned that if the lPFC was controlling attention to the emotional faces as we hypothesized, there should be evidence of a functional link between the control areas and the visual sensory association cortex (11). The demonstrated pattern of connectivity is consistent with our hypothesis that the lPFC regions identified in the main analysis are indeed influencing attention. Clearly, our PPI analysis alone cannot prove that lPFC controls activity in the fusiform cortex; the observed pattern of connectivity could equally well be produced by the fusiform controlling activity in the lPFC. However, our interpretation is in line with both the models of attentional control (10–12) and the more general understanding of the lPFC as providing a supervisory role in cognition (41).

Although we were able to demonstrate the predicted pattern of connectivity between lPFC and sensory cortex, we did not find an effect of attentional training on the gross activity of the face selective fusiform cortex (Supplement 1), which would have strengthened the interpretation of our results. While a single training session appears insufficient to individually demonstrate the effects of our intervention on every node of the attention circuit, future studies using longer training regimes may be able to show such an effect.

We had predicted that the rostral anterior cingulate cortex would be identified in our whole-brain analysis but did not find a significant effect. However, with a region of interest approach (Supplement 1), a small region of the rACC was found to display the same pattern of activity as the lPFC. Thus, it seems likely that the lPFC regions identified in our main analysis are one node of a larger control circuit that incorporates the rACC. It may also include the striatum, because our whole-brain analysis revealed bilateral striatal activity with a similar pattern of activity. We had not predicted these findings, so interpretation must be cautious; however, the striatum is a component of a well described circuit that includes the lPFC (42) and thus the striatal activity may reflect the efferent or afferent connections with the PFC.

We have suggested that attentional training may provide a model of one of the mechanisms involved in more complex psychological interventions such as CBT. Indeed, there is some evidence that CBT ameliorates the negative attentional biases found pretreatment in patients with anxiety (43). However, our study compared avoid-threat training and attend-threat training, either or both of which may actively influence attentional function. While this design provides the most sensitive measure as to which areas of the brain are influenced by attentional training, it cannot discriminate whether the observed effects result from the attend-threat training, the avoid-threat training, or both together. As the avoid-threat training is predicted to be therapeutic in anxiety (5,6,44,45), an interesting next step would be to assess the effects of this form of training in comparison with a control condition. Further, such a study could also incorporate an assessment of attentional function both before and after training, providing a more direct assay of the effect of the intervention on attention than the between-subject approach used in the current study.

A single session of attentional training was sufficient to tune lateral prefrontal function even when assessed using emotional stimuli of a completely different type (faces vs. words) to those employed in training. This generalization of training effect across stimulus type was also supported by the behavioral data, where a word-based training procedure influenced attention to pictures of faces. Interestingly, the effect of training was only evident when prototypical expressions of fear were used in the testing session, with no effects apparent when less intense facial expressions were employed. One interpretation of such results is that there is a threshold of emotional signal above which the training effect is manifested. Clearly, if attentional training is to be effective in clinical settings, it is important that it produces an effect on attention extending beyond the specific stimuli used in training, as demonstrated here.

The interpretation of studies that investigate treatment mechanisms in clinical groups can be confounded by factors other than exposure to the treatment. Thus, when treatments improve clinical state or significantly change behavior (e.g., [16,21,46]), there is an inevitable confounding of the treatment effects by variation in psychopathology (e.g., mood) or behavior (e.g., time spent looking at negative pictures). The design of our study minimizes such factors, first by studying a nonclinical population who did not experience a profound change of mood or anxiety, and second by using a behaviorally insensitive task during imaging such that the performances of the groups were equivalent. This allows a more straightforward interpretation of our results as the direct effect of attentional training. While it is important that these findings are extended to clinical groups, translational studies such as ours are well suited to demonstrating the basic neural mechanics underpinning treatment effects and for proof of concept in developing novel training strategies or specific psychotherapies.

In summary, the current study demonstrates that lateral prefrontal activity to emotional stimuli may be modified by a simple cognitive intervention known to alter attentional bias. This supports the proposal that modification of PFC function contributes to the effects of psychological interventions that target attentional processes and suggests that such interventions are mechanistically distinct from pharmacological approaches.

## Figures and Tables

**Table 1:** Demographic Details for Participants

Measure	Behavioral Assessment			Imaging Assessment		
	Avoid-Threat Training	Attend-Threat Training	*p*	Avoid-Threat Training	Attend-Threat Training	*p*
Female:Male	8:4	7:5	.67^a^	10:5	8:6	.6^a^
Age	21.4 (2.9)	24.3 (6.3)	.16	20.3 (.4)	20.5 (.5)	.64
BDI	2.8 (3.1)	2.6 (1.4)	.9	3.3 (.6)	3.1 (.5)	.75
STAI-Trait	31.2 (7.1)	31.9 (5.1)	.77	35.1 (1.5)	33.5 (1.5)	.39
STAI-State	27.1 (5.2)	28.8 (4.9)	.43	31.5 (2)	28.1 (1.6)	.21

All continuous measures are reported as mean (SD). BDI, Beck Depression Inventory; STAI, State-Trait Anxiety Inventory.

a Chi-square test was used to test the null hypothesis of no difference between groups. For all other measures, independent *t* tests were used.
