# Supplementary material for: Lateral Prefrontal Cortex Mediates the Cognitive Modification of Attentional Bias
Source: Biol Psychiatry. 2010 May 15;67(10):919–25. doi: 10.1016/j.biopsych.2009.10.031 (PMC2866253; doi:10.1016/j.biopsych.2009.10.031)
Supplement: Supplement 1 [file mmc1.pdf]

Supplemental Information

Supplementary Methods and Materials

Psychophysiological Interaction Analysis

The connectivity analysis was designed to assess whether the lateral frontal regions identified in the main analysis were influencing attention to the faces as reflected in visual sensory association cortex activity (1). This was achieved using psychophysiological interaction (PPI) analyses (2) to examine the pattern of correlation between activity in the lateral PFC (lPFC) clusters and face selective visual sensory cortex (see below for description of the localizer analysis used to identify face selective cortex). Separate analyses were used for the right and left lPFC clusters. In both of these analyses three regressors were included: the timeseries of the peak voxel from the lateral frontal cluster of the participant's fMRI data (the "physiological" regressor), a regressor coding for whether each block of 20 trials was attended or unattended (the "psychological" regressor) and a final regressor reflecting the interaction of the two (the "interaction" regressor). The interaction regressor provides a measure of how the correlation between activity in the lPFC and sensory cortex differs when attention is directed towards or away from the faces. The mean value of this interaction regressor was extracted from each participant's left and right fusiform masks, for both PPI analyses, resulting in four data points per participant.

Predicted Connectivity

The question addressed in this analysis was whether the pattern of connectivity between the identified lPFC clusters and the visual cortex was consistent with our hypothesis that lPFC activity controls attentional resources. Our specific prediction was based on two pieces of information: a) the pattern of activity in the lPFC as demonstrated in the main analysis and b) the predicted effect of training on sensory activity-- increased attention to particular stimuli

(such as fearful faces) as a result of training should increase the activity associated with those stimuli (1). In short, we asked whether the connectivity was such that a) would lead to b). As illustrated in Figure S1, the predicted effect emerges if there is a relatively more positive correlation between the control and sensory regions during unattended as compared to attended blocks. It is important to note that while our main analysis demonstrates that frontal *activity* differs between groups, the *connectivity* should not differ. In other words, we expect the lPFC to influence activity of the visual sensory cortex in both groups, with the attentional differences arising because the lPFC reacts to different stimuli in the two groups.

Localizer Procedure

The PPI analysis examined connectivity between the frontal regions identified in the main analysis and face selective sensory cortex. We used a localizer procedure (3) to identify the face selective regions separately in each participant. This was achieved by contrasting all trials in the faces task with all trials in a word based task which participants also completed in the same scanning session. The word task was of similar structure to the faces task (i.e., a centrally presented word was flanked by bars and presented for 200 ms) and allowed a separate localizer analysis to be performed for each participant. Specifically, we identified the peak voxel for the contrast of all faces > all words within both the left and right fusiform cortices (determined as > 50% probability of lying within the fusiform cortex using the Harvard-Oxford cortical atlas provided with FSL). Individual fusiform masks of 8 mm diameter were then created, centered on these co-ordinates.

Supplementary Analysis

In the main text of the article we report the results of a whole brain analysis of the effects of attentional training on the emotion x attention contrast. This identified bilateral striatal and lateral frontal activations. In addition to the lateral PFC, previous work has implicated both the rACC (4) and amygdala (5) as being involved in the control of attention to emotional stimuli. Given this prior evidence we reran the imaging analysis using a more sensitive region of interest (ROI) approach in order to assess for subtle effects of training missed on our main whole brain analysis.

Rostral Anterior Cingulate Cortex

The rACC ROI mask was constructed in standard space by including voxels which had a greater than 50% probability of lying in the ACC according to the Harvard-Oxford cortical atlas (included in the FSL package) and which had a y co-ordinate of > 30 (6). This analysis revealed a small cluster (*x y z* = 0 32 8, *Z*-max = 2.91, *p*-corrected = 0.04) in which activity associated with the emotion x attention contrast significantly differed between groups. Analysis of the mean extracted signal change associated with the fear-neutral contrasts confirmed that the pattern of this interaction was identical to that described in the lPFC clusters of the main analysis.

Amygdala

ROI masks for both left and right amygdala were defined in standard space as all voxels with > 50% probability of lying within the amygdala as defined by the Harvard-Oxford sub-cortical atlas. No significant activity was detected in either amygdala using the same analysis parameters as above.

Fusiform ROIs

In our PPI analysis we examine the connectivity between lPFC and individually defined fusiform regions of interest. When these ROIs were applied to the standard analysis there was evidence of greater activity during trials in which attention was directed towards the faces vs. those when attention was directed towards the bars [$F(1,27) = 6.7$, $p = 0.015$] as would be expected from face selective sensory cortex. There were no significant effects of training on gross activity [all $p > 0.1$].

The Role of Awareness of the Training Contingency

We have suggested that the training task used in this study alters “attentional bias” which implies that the acquired effects of the task do not require the conscious co-operation of participants. We note that explicit instruction can influence lateral frontal activation to emotional stimuli in other experiments (7). Therefore we examined whether participants might have become aware of the training contingency (i.e., that the probes always appeared behind the threatening/neutral word during training) and whether awareness of the contingency could explain the observed pattern of lateral frontal activation. Four participants in the avoid-threat group and 6 participants in the attend-threat group were found to have detected the contingency when explicitly asked at the end of the study [difference between groups not significant Fisher’s exact test, $p = 0.45$]. We therefore reran the initial analysis using only those participants who did not report awareness of the contingency (avoid-threat group $n = 11$; attend-threat group $n = 8$). Analysis was performed using “randomize”, the non-parametric statistical tool provided in FSL, as is appropriate when group sizes do not justify a parametric approach. A whole brain, cluster corrected analysis using the Feat default parameters (Z threshold = 2.3, $p < 0.05$) revealed a similar pattern of bilateral frontal and striatal activations in this smaller group indicating that awareness of the training contingency could not account for our effects.

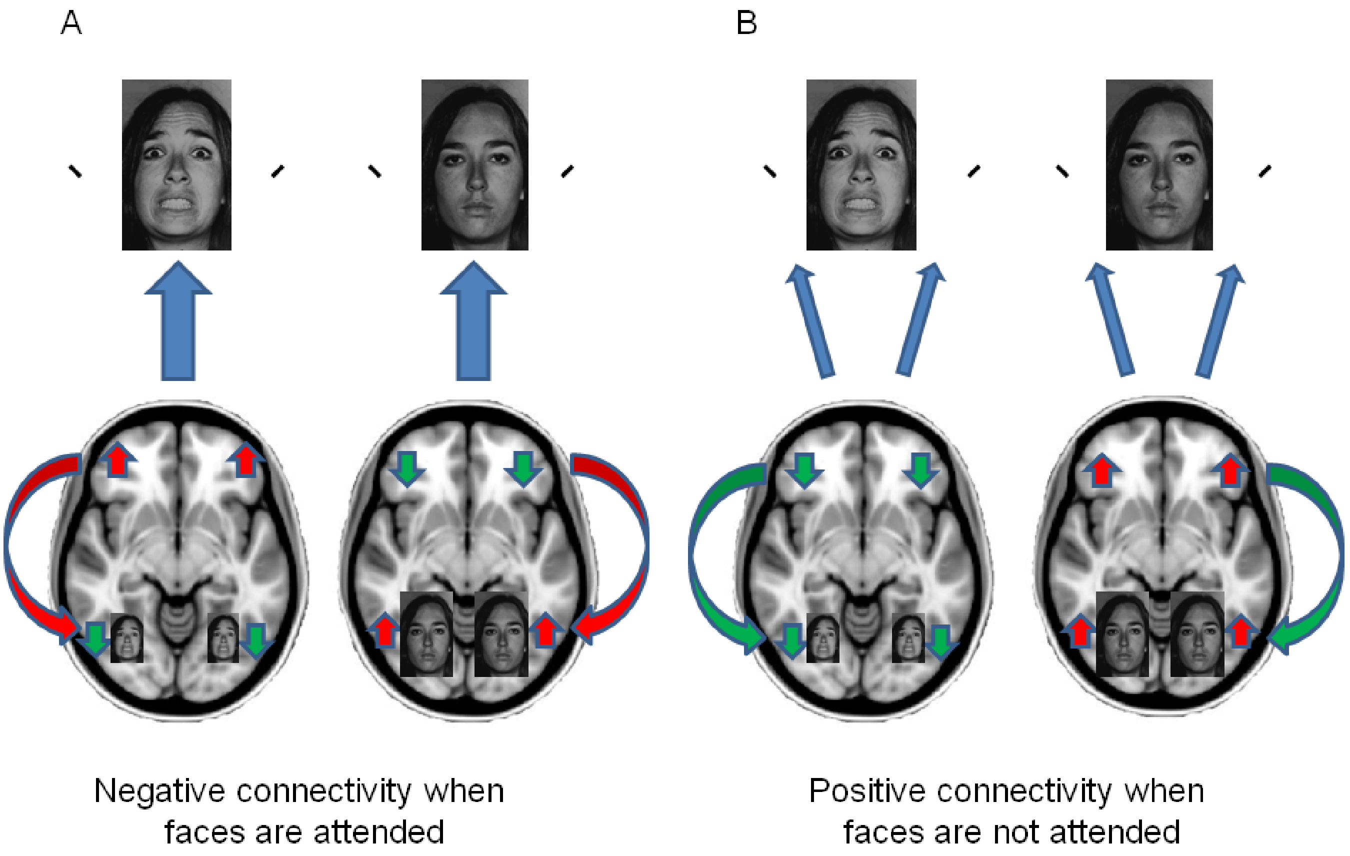

Figure S1. Predicted connectivity during the different trials of the faces-bars task. The task includes blocks of trials in which the participant responds to the face (A) and others in which the participant responds to the bars (B). Each block includes fearful and neutral faces which are depicted separately. The blue arrows indicate the direction of attention of the participant (towards the faces or the bars). Neural activity is represented by arrows inside the brain with: 1) The upper arrows representing the response of the lateral PFC as demonstrated in the main analysis (the illustrated response is that seen in the avoid-threat group). 2) The lower arrows representing the predicted response of face selective sensory cortex (avoid-threat training should favor the sensory representation of neutral rather than fearful faces). The crucial issue in the analysis is determining whether the connectivity between these areas is consistent with our hypothesis that activity in 1) leads to 2). In the diagram “connectivity” is illustrated by the curved arrows outside the brain (red arrow “negative connectivity”, green arrow “positive connectivity”). As can be seen, the expected pattern of sensory cortex activity emerges if the connectivity (i.e., correlation) between the regions is more positive in the bars trials (B) than the faces trials (A). The predicted pattern of connectivity in the attend-threat group is exactly the same; in this case the fearful faces will be favored as the lPFC response is reversed. Our PPI analysis tests for this predicted pattern of connectivity; specifically it assesses whether the correlation between the two areas is greater during the bars trials than during the face trials.

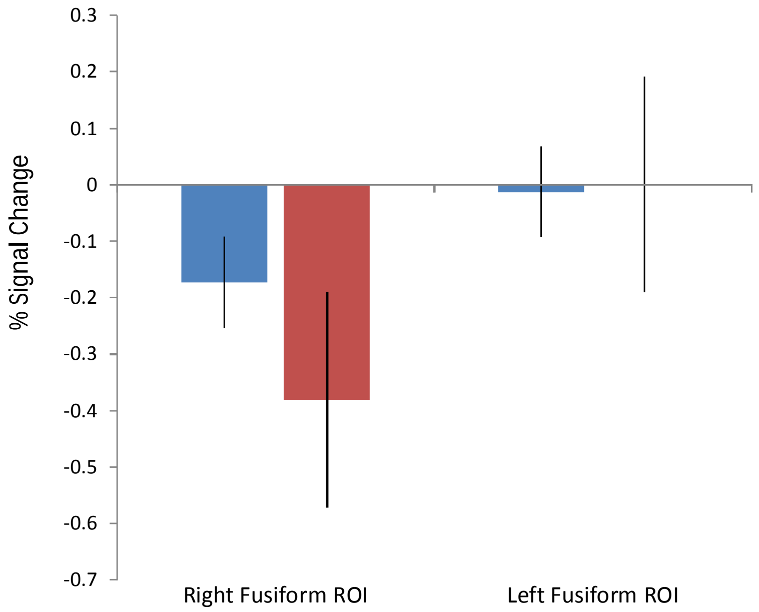

Figure S2. Mean (SEM) estimate of the signal change associated with the PPI regressors in the left and right fusiform cortex. Connectivity between the left (blue bars) and right (red bars) lPFC and left and right fusiform cortex was assessed using PPI analyses. The analyses were coded such that a positive PPI indicates a more positive correlation during faces trials whereas a negative value indicates a more positive correlation during bars trials—the hypothesized pattern. Although the results are more prominent within the right fusiform ROI, statistical analysis revealed a general negative value across all measures and did not justify separate analyses of left and right fusiform ROIs.

1. Vuilleumier P (2005): How brains beware: neural mechanisms of emotional attention. *Trends Cogn Sci* 9: 585-594.
2. Friston KJ, Buechel C, Fink GR, Morris J, Rolls E, Dolan RJ (1997): Psychophysiological and modulatory interactions in neuroimaging. *Neuroimage* 6: 218-29.
3. Kanwisher N, McDermott J, Chun MM (1997): The fusiform face area: a module in human extrastriate cortex specialized for face perception. *J Neurosci* 17: 4302-4311.
4. Bishop S, Duncan J, Brett M, Lawrence AD (2004): Prefrontal cortical function and anxiety: controlling attention to threat-related stimuli. *Nat Neurosci* 7: 184-188.
5. Bishop SJ, Duncan J, Lawrence AD (2004): State anxiety modulation of the amygdala response to unattended threat-related stimuli. *J Neurosci* 24: 10364-10368.
6. Bush G, Luu P, Posner MI (2000): Cognitive and emotional influences in anterior cingulate cortex. *Trends Cogn Sci* 4: 215-222.
7. Ochsner KN, Gross JJ (2005): The cognitive control of emotion. *Trends Cogn Sci* 9: 242-249.
